# Vitamin E Deficiency Secondary to Laparoscopic Sleeve Gastrectomy: A Case Report

**DOI:** 10.7759/cureus.83822

**Published:** 2025-05-09

**Authors:** Mohamed S Ahmed, Mariam Sandal, Ali Yammahi

**Affiliations:** 1 Department of General Surgery, Rashid Hospital, Dubai Health, Dubai, ARE

**Keywords:** bariatric surgery, case report, obesity, sleeve gastrectomy, vitamin e deficiency

## Abstract

Obesity is a prevailing health concern, with bariatric surgery being a common treatment option despite the potential nutritional risks and poorly understood postoperative physiological changes, particularly in the gastrointestinal tract. Neurological complications following bariatric surgery, often secondary to micronutrient deficiencies like vitamin B12, thiamine, and copper deficiencies, have been increasingly reported. This report presents a case of a 20-year-old female patient who developed neurological deficits, specifically bilateral lower limb weakness, following laparoscopic sleeve gastrectomy. Investigations revealed deficiencies in vitamin E, D, and folic acid. Despite the rarity of vitamin E deficiency post-sleeve gastrectomy, this deficiency led to bilateral axonal sciatic mononeuropathies, which improved with appropriate supplementation and supportive care. This case highlights the importance of regular nutritional monitoring and supplementation following bariatric surgery to prevent rare yet serious complications such as vitamin deficiencies leading to neurological disorders. Routine follow-ups and personalized care are essential for mitigating the risks of post-surgical deficiencies and improving patient outcomes.

## Introduction

Obesity, a surging health concern, with multifactorial causes, has inadvertently led to the increasing use of metabolic and bariatric surgery (MBS). Despite bariatric surgery being an effective therapeutic approach to obesity and its dire comorbidities, the nutritional risks accompanying it often go unaddressed, and the postoperative physiological changes to the gastrointestinal tract remain poorly understood [1]. MBS is leading to a rise in neurological complications, making it imperative to report and address this issue [2]. These complications can stem from mechanical issues such as strictures, or inflammation, but are primarily due to deficiencies in micronutrients such as vitamin B12, thiamine, and copper. They are often linked to the type of procedure performed, with a higher prevalence in malabsorptive procedures, resulting mainly in a deficiency in fat-soluble vitamins, and often occur within a certain time span of the procedure. However, vitamin E deficiency is neither a known nor common entity seen following sleeve gastrectomy (SG) [3].

Close nutritional monitoring is essential for MBS patients, with regular checks at intervals of six weeks, starting at 3, 6, and 12 months postoperatively. This can then be followed by annual checks and fortified with lifelong multivitamin supplementation. It is imperative to address the nutritional needs of patients undergoing MBS, and awareness of both common and uncommon neurological complications is crucial [4].

In this study, we report a case of a young female patient who underwent laparoscopic SG, and developed neurological deficits postoperatively, attributed to vitamin E deficiency.

## Case presentation

A 20-year-old otherwise healthy woman (BMI 55.71) was admitted to undergo elective laparoscopic SG following multiple failed attempts at weight loss through lifestyle changes. The patient tolerated the procedure and had an uneventful postoperative course. However, a month later, she presented to the Emergency Department with a one-week history of generalized abdominal pain, frequent vomiting, and decreased appetite. There was otherwise no fever, changes in bowel or urinary habits reported.

**Figure 1:**
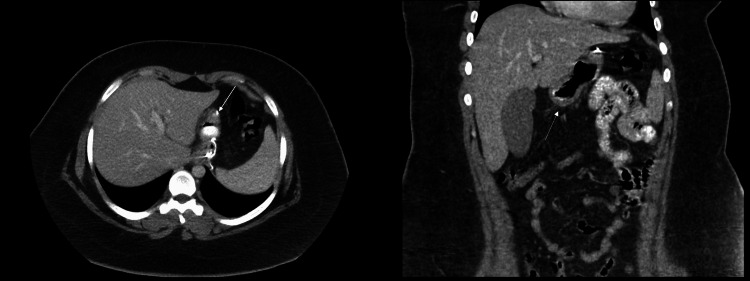
CT of the abdomen and pelvis with intravenous and oral contrast (axial and coronal views) Arrows show a healthy sleeve stomach with an intact staple line, without evidence of any extraluminal leak of oral contrast. No other pathology was noted; there was normal enhancement of the gastric tissue.

CT of the abdomen and pelvis with IV and oral contrast (Figure 1) was ordered, as per the bariatric protocol at our facility, to rule out a multitude of etiologies such as postoperative leak, bleeding, twist, and strictures. No abnormalities were detected.

**Figure 2:**
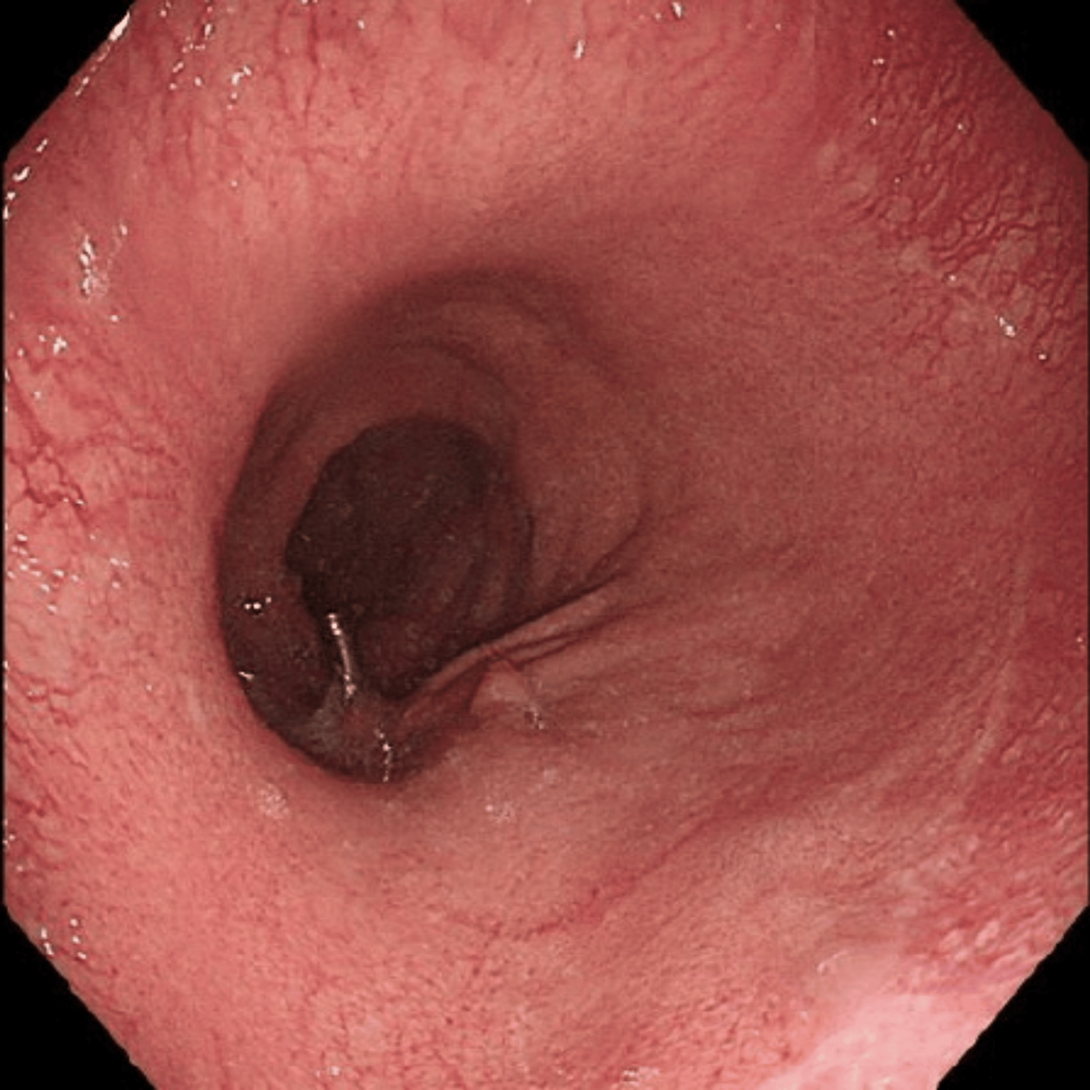
Upper gastrointestinal endoscopy (gastroscopy) It showed scattered erythematous patches along the gastric mucosa; however, no ulcers or mucosal atrophy was seen.

Following this, the patient underwent upper gastrointestinal endoscopy (Figure 2) that revealed an incompetent lower esophageal sphincter and acute angulation at the incisura. The patient was admitted and supportive care was initiated, including hydration, analgesia and antiemetics. Her condition improved throughout her hospital stay and she was subsequently discharged.

However, the patient returned to the Emergency Department three months following her laparoscopic SG, with a seven-day history of abdominal pain, nausea, and multiple episodes of vomiting. The pain was generalized and exacerbated by oral intake. This was accompanied by a two-week history of bilateral lower limb weakness and numbness, along with dizziness. On examination, the patient was noted to be tachycardic, with a rather soft and non-distended abdomen. Upon further questioning, the patient noted that she was compliant with the medications and vitamins prescribed on discharge.

The patient was then admitted and underwent further investigations that revealed low levels of vitamin E, D, and folic acid -- 0.27 mg/L (reference 5.0-18.0 mg/L), 10.2 ng/mL (30-100 ng/mL) and 2.0 ng/mL (4.6-34.8 ng/mL), respectively, as displayed in Table 1.

**Table 1:** Laboratory work-up

Parameter	Patient’s value	Reference range
Vitamin E	0.27 mg/L	5.0-18.0 mg/L
Vitamin D	10.2 ng/mL	30-100 ng/mL
Folic acid	2.0 ng/mL	4.6-34.8 ng/mL

A nerve conduction study was also ordered in view of the aforementioned bilateral weakness, which revealed bilateral axonal sciatic mononeuropathies with predominant involvement of peroneal components. The possibility of bilateral common peroneal mononeuropathies was also considered.

Vitamin replacement and supportive care were initiated; vitamin E was taken orally, 600 mg, twice a day. Throughout her hospital stay, the patient remained stable and was able to ambulate with support. The patient was then discharged with follow-up in the Neurology Clinic, as well as Physiotherapy.

## Discussion

The wide dissemination of MBS has inadvertently resulted in neurological complications, primarily secondary to deficiencies in certain nutrients and vitamins. Rapid loss of weight is often secondary to the malabsorption of vital nutrients, but the swift progress experienced by patients may obscure the potential harmful metabolic derangements that could ensue. Bariatric surgery becoming more popular mandates closer follow-up and proper nutritional support to prevent undesired nutritional deficits, such as the complications highlighted in this case following said surgeries.

Chronic peripheral neuropathies are the most frequently reported neurological complication following bariatric surgery. They can be further classified into mononeuropathy, sensitive-motor polyneuropathy, and radiculoplexopathy. While typical and anticipated in patients who underwent surgery many years ago, they can also occur in the short term, as demonstrated in several studies conducted in South America, where these neuropathies were also commonly encountered [5-7].

Likely underestimated due to some cases being subclinical, the range of reported neurological complications following malabsorptive MBS was found to be 4.6% to 16% [8]. A wide range of neuropathies have been elicited in the literature, including Guillain-Barré syndrome, Wernicke's encephalopathy, and several other cranial nerve palsies, and myelopathies. In addition to the classical above-mentioned complications, one study in Brazil showcased three instances of motor neuron disease and five of central nervous system demyelination that were most likely potential complications of MBS [7,9]. While establishing a direct cause-and-effect relationship in these cases is challenging, these findings may go beyond mere coincidence [7,10].

An impaired immune response, along with oxidative stress and neuronal disorders, has been associated with severe vitamin E deficiency. Those at the highest risk of developing fat-soluble vitamin deficiencies are those who underwent malabsorptive procedures, although the clinical manifestations secondary to vitamin E deficiency are rarely reported and documented. Unfortunately, the data available on vitamin E statuses among MBS patients is scanty. It is yet to be elucidated that following MBS, vitamin E supplementation required to either prevent or treat deficiencies requires optimal dosing, which is yet not established in the recent literature. Further studies are necessary to address the gaps in knowledge [11]. As delineated by the United States National Academy of Sciences Food and Nutrition Board, the recommended dietary allowance (RDA) for vitamin E for adolescents and adults is 15 mg of dietary alpha-tocopherol [5].

Although neurological complications primarily arise due to micronutritional deficiencies, they could also be secondary to mechanical or inflammatory mechanisms. Vitamin B12, thiamine, and copper constitute the most commonly reported deficiencies [11]. Not only do neurological complications manifest at relatively predictable time points following MBS, but they are also closely associated with the specific surgical procedure performed as highlighted earlier. In the early postoperative period, patients may experience compression/stretch-induced peripheral nerve injury, inflammatory polyradiculoneuropathy, or rhabdomyolysis [6]. On the other hand, long-term complications, developing months and years postoperatively, could include hypocupric myelopathy, and combined system degeneration that is often the result of deficiency in vitamin B12. MBS patients need thorough nutritional monitoring, including routine assessment of micronutrients at 6 weeks, and 3, 6, and 12 months postoperatively, and then annually thereafter, along with lifelong multivitamin supplementation. Ongoing vigilance for both common and rare neurological complications is crucial [12-14].

There is inadequate evidence to justify regular screening for deficiencies in essential vitamins, namely, vitamin E and K. However, routine screening for vitamin A levels is recommended following malabsorptive MBS procedures like biliopancreatic diversion with duodenal switch (BPD/DS) or BPD, as vitamin A deficiency can manifest as ocular complications. In such cases, supplementation of vitamin A, either alone or with other fat-soluble vitamin, could be necessary [15].

## Conclusions

Our patient, following laparoscopic SG, suffered from symmetrical sciatic and peroneal nerve mononeuropathies secondary to nutritional deficiencies, particularly of vitamin E. This is an uncommon complication following MBS, especially SG. To the best of our knowledge, there is little to no data describing the aforementioned complication, and the cause often is attributed to malabsorptive procedures, rather than restrictive ones as seen in this case.

The clinical presentation differs among patients, and hence, demands personalized work-up as the manifestations will vary, depending on multiple factors such as inflammatory process, local compression, and numerous nutritional deficiencies. Preventative measures are both feasible and imperative to mitigate peripheral nerve complications following MBS.
